# Human Vagus Nerve Branching in the Cervical Region

**DOI:** 10.1371/journal.pone.0118006

**Published:** 2015-02-13

**Authors:** Niels Hammer, Juliane Glätzner, Christine Feja, Christian Kühne, Jürgen Meixensberger, Uwe Planitzer, Stefan Schleifenbaum, Bernhard N. Tillmann, Dirk Winkler

**Affiliations:** 1 Institute of Anatomy, University of Leipzig, Faculty of Medicine, Leipzig, Germany; 2 Department of Neurosurgery, University Clinic of Leipzig, Faculty of Medicine, Leipzig, Germany; 3 Department of Cardiology, University Clinic of Leipzig, Faculty of Medicine, Leipzig, Germany; 4 Department of Orthopedic, Trauma and Reconstructive Surgery, University Clinic of Leipzig, Leipzig, Germany; 5 Institute of Anatomy, University of Kiel, Kiel, Germany; University of California, Los Angeles, UNITED STATES

## Abstract

**Background:**

Vagus nerve stimulation is increasingly applied to treat epilepsy, psychiatric conditions and potentially chronic heart failure. After implanting vagus nerve electrodes to the cervical vagus nerve, side effects such as voice alterations and dyspnea or missing therapeutic effects are observed at different frequencies. Cervical vagus nerve branching might partly be responsible for these effects. However, vagus nerve branching has not yet been described in the context of vagus nerve stimulation.

**Materials and Methods:**

Branching of the cervical vagus nerve was investigated macroscopically in 35 body donors (66 cervical sides) in the carotid sheath. After X-ray imaging for determining the vertebral levels of cervical vagus nerve branching, samples were removed to confirm histologically the nerve and to calculate cervical vagus nerve diameters and cross-sections.

**Results:**

Cervical vagus nerve branching was observed in 29% of all cases (26% unilaterally, 3% bilaterally) and proven histologically in all cases. Right-sided branching (22%) was more common than left-sided branching (12%) and occurred on the level of the fourth and fifth vertebra on the left and on the level of the second to fifth vertebra on the right side. Vagus nerves without branching were significantly larger than vagus nerves with branches, concerning their diameters (4.79 mm vs. 3.78 mm) and cross-sections (7.24 mm^2^ vs. 5.28 mm^2^).

**Discussion:**

Cervical vagus nerve branching is considerably more frequent than described previously. The side-dependent differences of vagus nerve branching may be linked to the asymmetric effects of the vagus nerve. Cervical vagus nerve branching should be taken into account when identifying main trunk of the vagus nerve for implanting electrodes to minimize potential side effects or lacking therapeutic benefits of vagus nerve stimulation.

## Introduction

Vagus nerve stimulation (VNS) is becoming an increasingly popular therapy for patients with refractory epilepsy [1–8] and psychiatric conditions such as depression [9,10]. Chronic heart failure became another field of application [11,12]. For the treatment using VNS, an electrode is placed around the cervical vagus nerve (CVN). The electrodes are either positioned on the left side, e.g. for the therapy of epilepsy, or on the right side, e.g. to treat cardiac dysfunction. The implantation procedure itself appears to be fairly easy on basis of published data on the CVN. It is assumed to run lateral and parallel to the common carotid artery and with branching only in a very small number of cases [13–15]. Against this widely accepted postulate, we experienced CVN branching as a common phenomenon when dissecting this anatomical region in body donors.

Though CVN branching has not yet been described as a source of error during the implantation of VNS electrodes, it is obvious that electrode placement at a different site than the main trunk of the CVN might explain some of the side effects or insufficient therapeutic effect of VNS. Undesirable side effects such as voice alterations, dyspnea [6,9,16], vocal cord palsy [16], neck and throat pain and coughing have been described in 17% of the patients treated with VNS [6]. Beyond that, the symptom improvement related to VNS is inconsistent. A majority of the patients with VNS results in major improvements and sometimes complete recovery from the symptoms, but VNS fails completely in approximately 25% of all cases [5]. These findings might also be related to structural anomalies of the CVN [17].

Previously, the CVN has mostly been described in an anatomical context but not in a surgical one. Published anatomical data consider the CVN and its branches to be structures of risk rather than a target of surgical intervention [17–19]. Furthermore, anatomical and physiological findings in animals can hardly be transferred to human patients that undergo VNS, since the human CVN anatomy has been described to be divergent from feline [20], canine [21] and rabbit CVN [22,23] concerning its histological composition.

Taking the issues of a potential CVN branching and the lack of anatomical data of humans into account, we aimed at investigating the prevalence of CVN branching. We therefore accessed the CVN in the carotid sheath of post-mortem body donors in the same manner and to the same extent of the surgical exposure as done with patients that undergo surgical electrode implantation for VNS. We traced these branches back, but only within the carotid sheath. Surprisingly, CVN branching was found in almost one third of the donors.

## Materials and Methods

### CVN preparation

The course of the CVN in the region of the carotid triangle was dissected in 35 human donors (21 females, 14 males), on the left side in 3 cases, on the right side in one case and bilaterally in 31 cases, accounting for 66 CVN in total (Table 1). The mean age of the donors was 87.23 ± 6.72 years (range 69 to 103 years). Institutional approval was obtained. While alive all body donors gave their informed and written consent to the donation of their bodies for teaching and research purposes. Being part of the body donor program regulated by the Saxonian Death and Funeral Act of 1994 (third section, paragraph 18 item 8), institutional approval for the use of the post-mortem tissues of human body donors was obtained from the Institute of Anatomy, University of Leipzig. The authors declare that all experiments have been conducted according to the principles of the Declaration of Helsinki. Anatomical fixation and conservation of the body donors was accomplished with ethanol-glycerin according to our house protocol [24,25].

**Table 1:** Baseline characteristics such as donors’ age and gender are given and the extent to which the vagus nerve could be visualized in vertebral segments.

Number	Age	Gender	Preparation from level to level				Branching (level)	
	[years]		Left		right		left	right
								
1	97	♂	C5 cr—C6 cd	1.5	C5 cr—C7 cd	2.5		
2	80	♀	C5 cr—C6 cd	1.5	C5 cr—C7 cr	2.0		
3	91	♀	C3 cd—C6 cd	3.0	C4 cd—C6 co	1.8		
4	89	♀	C4 co—Th1 cr	2.8	C5 cd—Th1 co	2.8		
5	84	♂	C6 cr—Th1 cr	2.0	C3 cr—C5 cd	2.5		C3 cd
6	76	♂	C5 cd—C7 cd	2.0	C4 co—C6 cd	2.3		
7	90	♀	C3 co—C7 co	4.0	C2 cr—C6 cr	4.0	C4 cd	C2 cd
8	83	♂	C4 cd—C6 cr	1.5	C3 cd—C5 cd	2.0		
9	84	♀	C3 cr—C7 cr	4.0	C3 cd—C7 cd	4.0		
10	91	♀	C5 co—Th1 co	3.0	C5 cd—Th1 cd	3.0		
11	87	♀	C6 cd—Th1 cd	2.0	C4 cd—C6 cd	2.0		
12	87	♂	C3 cd—C5 cd	2.0	C4 cr—C6 cd	2.5		C4 co
13	87	♀	C4 cd—C7 cr	2.5	C5 cr—C6 co	1.3		C5 co
14	69	♂	C5 cd—C7 cd	2.0	C5 co—C7 co	2.0		
15	86	♀	C4 co—C5 cd	1.3				
16	91	♀			C5 cd—C7 cd	2.0		
17	80	♂	C3 cd—C5 cd	2.0				
18	97	♀	C4 cr—C5 cr	1.0				
19	90	♀	C5 co—C7 co	2.0	C4 co—C6 cr	1.8		
20	81	♀	C3 co—C6 cd	3.3	C4 cr—C7 cd	2.5	C5 cr	
21	94	♂	C3 co—C5 cd	2.3	C4 cr—C6 co	2.3		C5 cr
22	73	♀	C5 cr—Th1 cr	2.0	C5 co—Th1 co	3.0	C5 co	
23	86	♀	C3 cd—C6 co	2.8	C3 cr—C5 cd	1.5		
24	90	♀	C4 co—C5 cd	1.3	C4 cr—C6 cd	2.5		C4 cd
25	86	♂	C5 co—Th1 cr	2.8	C4 co—C6 cd	2.3		
26	88	♂	C6 cr—C7 cd	1.5	C5 cd—C7 cd	2.0		
27	89	♀	C5 cr—C7 cd	2.5	C5 co—C7 cd	2.3		C5 co
28	87	♂	C4 cr—C7 co	3.3	C4 co—C7 cd	3.3		
29	92	♂	C5 cr—C6 cr	1.0	C4 cd—C6 co	1.8		
30	90	♂	C5 cd—C7 co	1.8	C4 co—C6 cr	1.8		
31	94	♀	C4 cr—C6 co	2.3	C3 co—C5 co	2.0		
32	81	♀	C4 cd—C6 cd	2.0	C5 cr—C7 cr	2.0		
33	103	♀	C1 cd—C4 cd	3.0	C2 cr—C4 cd	2.5		
34	90	♂	C4 cd—C7 cd	3.0	C6 cr—Th1 co	2.3	C5 cr	
35	90	♀	C3 cr—C5 cr	2.0	C3 co—C5 co	2.0		
**Range**			C1—Th1	1.0–4.0	C2—Th1	1.3–4.0	C4—C5	C2—C5
**Mean**				2.3		2.3		
**Summary**	**87.23**	**♀/♂**	**34**		**32**		**4**	**7**
	**± 6.72**	**21/14**						

Also, the height of vagus nerve branching is documented. C = cervical spine, Th = thoracic spine, cd = caudal base plate, co = center of the vertebral corpus, cr = cranial base plate.

A similar surgical exposure was performed as done when implanting VNS electrodes in patients to treat chronic heart failure or epilepsy [26]. Following a careful incision of the skin, the platysma was transected sharply along the medial border of the sternal head of the sternocleidomastoid muscle. The incision was oriented in an oblique manner, which is different from the exposure described by Spuck and coworkers [26]. The incision started on the level of the laryngeal prominence and was extended caudally towards the superior belly of the omohyoideus muscle. Cranially, the incision was extended until the posterior belly of the digastric muscle was reached. The superficial layers of the cervical fascia were bluntly dissected with scissors [27]. The exposed site was held open by a spreader. Care was taken not to transect the nerves of the ansa cervicalis [28]. If necessary, the anterior jugular vein was partly removed. Following this, the carotid sheath was opened ventrally until the internal or common carotid artery was visualized, followed by the internal jugular vein. Identification and preparation of the CVN was done at the full length of the surgical exposure in the carotid triangle. Potential CVN branches were traced back macroscopically in the carotid sheath.

### X-ray, casting and histology samples

Metal needles were used to mark the most cranial and caudal part that was visible of the CVN and CVN branches before X-rays were taken from the cervical region in the anterior-posterior and lateral projection (Ziehm Vision RFD, Ziehm Imaging GmbH, Nuremberg, Germany). The position of the metal needles was either attributed to the cranial or caudal baseplate, or to the corpus of the respective vertebra. Distances between two baseplates were defined as 0.5 vertebral segments and distances between a baseplate and the corpus as 0.25 vertebral segments.

Two tissue samples were obtained from each CVN and the respective branches. The first pair of tissue samples was used for determining the nerve’s cross-sections after casting with polyvinyl siloxane (HS-A silicon; Henry Schein Inc., Melville, NY, USA), according to [29]. The casts of the CVN were scanned at 1200 dpi before calculating their largest diameter (d) the cross-sectional area (c) with Datinf Measure software (Datinf GmbH, Tübingen, Germany). The other tissue samples were immediately dehydrated in ascending ethanol series and then embedded with paraffin. Serial sections of 15 μm were stained with hematoxylin-eosin (HE). Three investigators (NH, JG, UP) scanned for the presence of nerve fibers. To this end, at least three slices with ten randomly selected fields were investigated under 100-fold magnification from each specimen [30].

### Statistical analysis

SPSS version 20.0 (Armonk, NY, USA) was used for statistical evaluation. Normal distribution was determined with the Kolmogorow-Smirnow test. The chi-squared test was applied to determine gender- or side-related differences in the occurrence of CVN branching. Comparison of the CVN diameters and cross-sectional areas was accomplished with the Student’s t-test. *P-values* of 0.05 or less were considered as statistically significant.

## Results

### CVN branching

**Fig 1:**
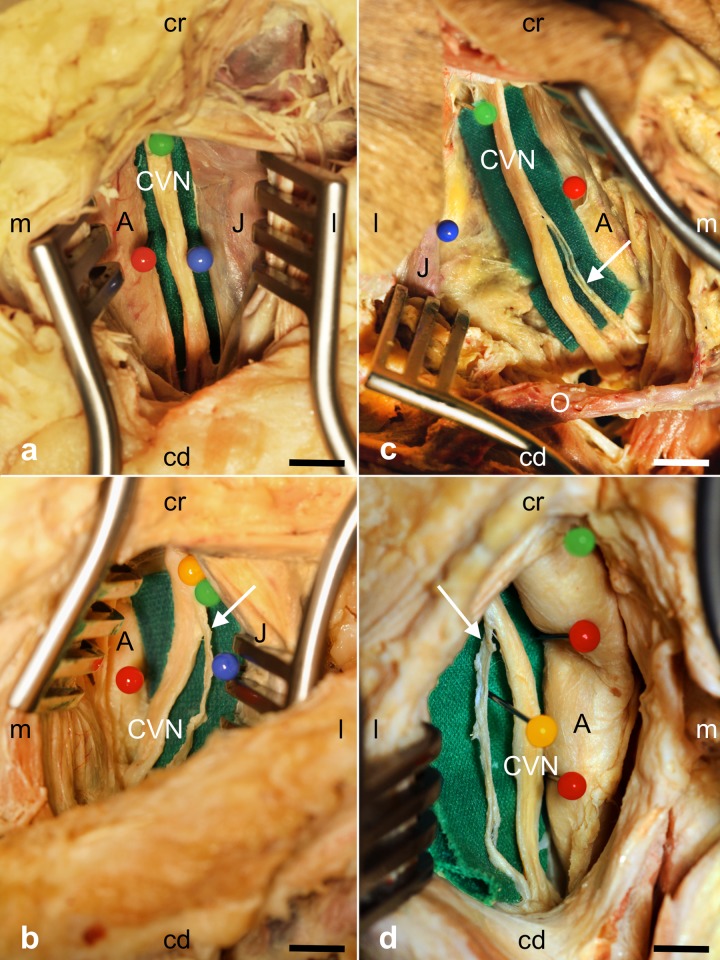
Images taken during dissection of the cervical vagus nerve (CVN) in the carotid sheath. Fig. 1a shows a left-sided CVN without branching and Fig. 1c-d CVN with branches on the left side (1b) or on the right side (1c,d). Arrows indicate the branches. C = (common or internal) carotid artery, J = internal jugular vein, O = superior venter of the omohyoideus muscle; cd = caudal, cr = cranial, m = medial, l = lateral; scale bar = 15 mm (a,b), 12 mm (c,d).

**Fig 2:**
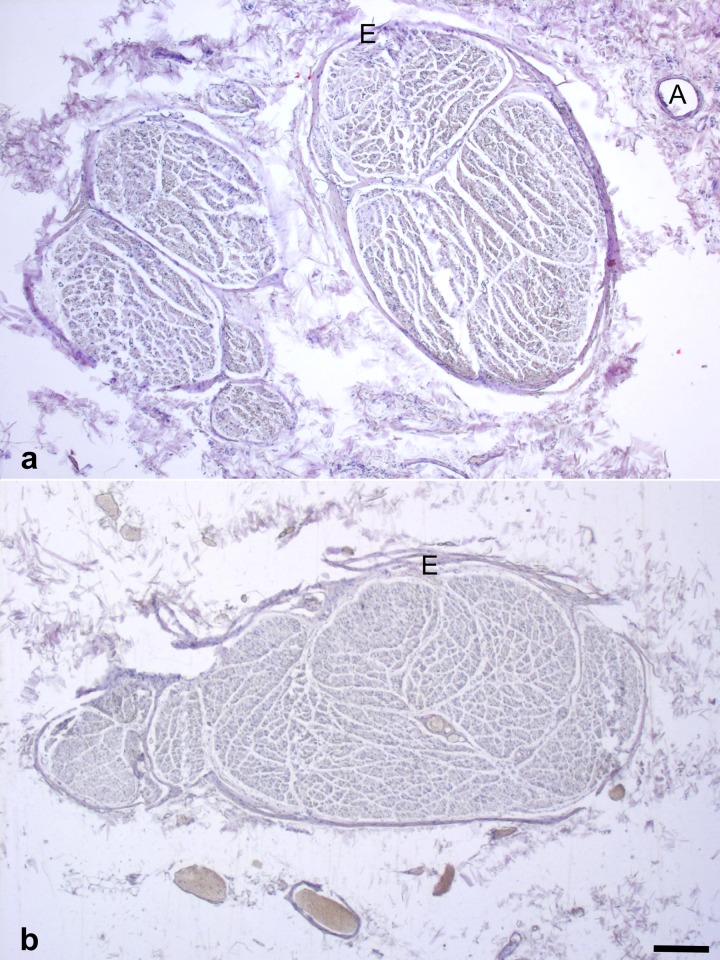
Hematoxylin-eosin stained histology samples obtained from the vagus nerve (2a) and from a vagus nerve branch (2b) for evaluating the existence of nerve fibers. A = arterial branch from the inferior thyroid artery, E = epineurium; scale bar = 500 μm.

Branching of the CVN in the carotid sheath was observed in 29% of the donors (10/35), in 26% (9/35) unilaterally and in 3% (1/35) bilaterally (Fig. 1; Table 1). Left-sided branching of the CVN was less common than branching on the right side with 12% (4/34 on the left) and 22% (7/32 on the right), respectively. These branches extended to the inferior larynx and to the upper mediastinum. Histological analysis confirmed nerve fibers in 100% of the CVN samples (66/66) and in 100% of the CVN branches (11/11; Fig. 2).

### CVN and branch diameters and cross-sections

The mean values of the diameters and cross-sectional areas of the left CVN (d = 4.65 mm; c = 7.04 mm^2^) did not vary significantly from the right ones (d = 4.59 mm; c = 6.74 mm^2^; Table 2). Also, the mean diameters and cross-sectional areas did not vary significantly between females (d = 4.43 mm; c = 6.31 mm^2^) and males (d = 4.90 mm; c = 7.74 mm^2^). However, significantly larger diameters (d = 4.79 mm vs. d = 3.78 mm; p = 0.015) and cross-sectional areas (c = 7.24 mm^2^ vs. c = 5.28 mm^2^; p = 0.045) were found when comparing the CVN without to those CVN with branches.

**Table 2:** Statistical comparison of vagus nerve diameters and cross-sections (mean value ± standard deviation).

Vagus nerve	left			right			*p-value*
							
diameter [mm]	4.65	±	1.26	4.59	±	1.30	*0*.*840*
cross-sectional area [mm^2^]	7.04	±	3.36	6.74	±	2.46	*0*.*678*
							
	**female**			**male**			
diameter [mm]	4.43	±	1.12	4.90	±	1.43	*0*.*136*
cross-sectional area [mm^2^]	6.31	±	2.57	7.74	±	3.27	*0*.*051*
							
	**no branching**			**branching**			
diameter [mm]	4.79	±	1.30	3.78	±	0.68	*0*.*015*
cross-sectional area [mm^2^]	7.24	±	3.04	5.28	±	1.60	*0*.*045*
							

### Extent of the surgical exposure and cervical levels of CVN branches

**Fig 3:**
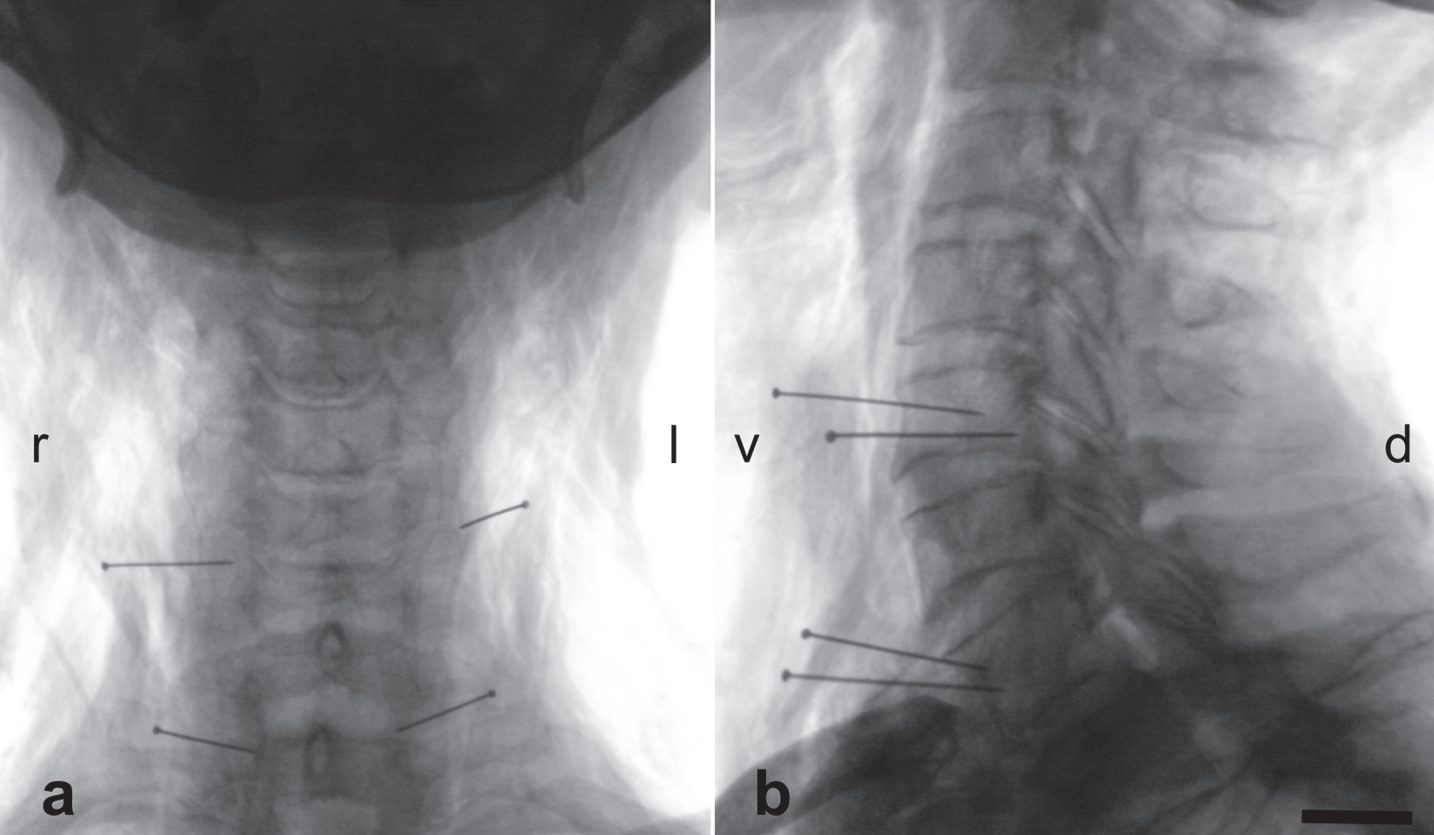
X-rays obtained from the cervical spine of a 69 year-old male in the anterior-posterior (3a) and in the lateral projection (3b). Metal needles indicate the most cranial and caudal part of the vagus nerve that could be visualized with the surgical approach to the carotid triangle. d = dorsal, l = left, r = right, v = ventral; scale bar = 10 mm.

The maximum extent of CVN exposure in the carotid sheath ranged from 1.0 to 4.0 cervical vertebra levels on the left side (mean 2.3 levels) and from 1.3 to 4.0 levels on the right side (mean 2.3 levels), indicated by the X-rays (Fig. 3; Table 1). Branching on the left-sided CVN was mostly observed between the fourth and fifth cervical vertebra and between the second and fifth cervical vertebra on the right side (Table 1).

## Discussion

### Are CVN branches possibly at risk of being stimulated during vagus nerve stimulation?

We show that CVN branching in the carotid sheath is considerably more frequent than described previously. CVN branches were found in 29% of the body donors (Fig. 1; Table 1). The vagus nerve is a complex and functionally most relevant cranial nerve concerning nerve stimulation. Meanwhile the relevance of the CVN has changed from a structure at risk to a stimulation target site in VNS. Existing studies mostly describe the CVN in an anatomical context [13–15,31,32], not in a surgical one. The vagus nerve and its branches such as the recurrent or inferior laryngeal nerve were also identified as structures of risk in other surgical procedures such as thyroidectomy [17–19].

The vagus nerve has been described to run lateral [13–15] or ventrolateral [33] and parallel to the carotid arteries, following the aortic arch on the left and the subclavian artery on the right side, with the internal jugular vein lying lateral or posterior to it [28,34]. The vagus nerve supplies branches to the facial [35], the glossopharyngeal [36], the accessory [37] and the hypoglossal nerve [38] in some cases before entering the carotid sheath. Also, connections to the cervical sympathetic trunk have been described [39–41] for the vagus nerve after entering the thorax and for the recurrent laryngeal nerve [42,43]. However, CVN branching in the carotid sheath has previously been shown to be an extremely rare variation [14,32], associated with embryonic vascular abnormalities such as a lusory artery [3,44]. These studies identified the CVN branches as the non-recurrent laryngeal nerve, with a prevalence ranging from 0.3% to 1% [3,45]. In contrast, we showed unilateral CVN branching in 26% and bilateral branching in 3% of all cases (Table 1). HE histology proves that the suspected branches consist of nerve fibers in all cases (Fig. 2). CVN branching occurs more often on the right than on the left side, confirming the findings of Coady and coworkers [3]. The side-dependent differences in CVN branching may be linked to the asymmetric effects of the vagus nerve [46], with the left one mainly connected to the atrioventricular node and the right one to the sinuatrial node [47]. Side-dependent differences in sympathetic and parasympathetic and the effector organs were however not the aim of this study.

There are no gender- or side-related differences in the diameters or cross-sections of the CVN (Table 1), as it is the case for the recurrent laryngeal nerve [48]. However, significantly larger diameters and cross-sections are found for the CVN without cervical branching than for the CVN with branching, indicating that there is a functional separation of CVN that comes with the branching. CVN branches might erroneously be attributed to the main trunk of the CVN or mistakenly become transected with serious consequences for the patient, e.g. in case of a non-recurrent laryngeal nerve. Another interesting finding is the arterial branch originating from the inferior thyroid artery, supplying the main trunk of the vagus nerve. This branch is seen in a majority of the cases in gross dissection (unpublished results) and in the histology samples (Fig. 2), confirming the findings of Fernando and coworkers [49]. Loss of blood supply to the CVN from this artery, e.g. after thyroidectomy, is hypothesized to cause disorders of motor speech [49].

From our findings in macroscopic dissection, we identify a small nerve diameter or cross-section as a risk when implanting the electrode at the wrong site. Furthermore, the suspicion of CVN branching could be verified by means of X-ray intraoperatively. We show that branching is found on the level of the forth and fifth cervical vertebra on the left side and on the level of the second to fifth cervical vertebra on the right side (Fig. 1; Table 1). These findings may help minimize the risk of stimulating CVN branches as the wrong stimulation sites.

### Are CVN branches a possible explanation for the side effects or the missing therapeutic efficiency of VNS?

VNS has been shown to successfully attenuate or stop seizures [7,8], to decrease hospitalization time [4], unexpected death related to epilepsy [50] and to improve the quality of life in epilepsy patients [47]. The seizure-reducing effects seem to be related to the afferent projections of the vagus nerve to the thalamus, limbic system [9,10,51,52], the solitary tract and the locus coerulus [53]. Large reviews on VNS in epilepsy show that the mean seizure reduction rates range from 45% [5] to more than 50% [2]. Complete remission rates are observed in 6% and 27% of the patients [3,6]. However, approximately 25% have no therapeutic benefit from VNS [5]. These observations might be related to the anatomy of the CVN in the carotid sheath and to the branching patterns to the effect that stimulation of CVN branches results in insufficient therapeutic effect. Surgery-related side effects such as voice alterations, dyspnea [6,9], vocal cord palsy [16], coughing, neck and throat pain [6] are found in 17% of the patients that undergo VNS [16,54]. Some of these complications may probably be attributed to branches of the vagus nerve, especially the superior and to the (non-) recurrent inferior laryngeal nerve. However, the left-sided vagus nerve is the most common stimulation site to treat epilepsy or psychiatric conditions such as depression [9,10]. Here, branching was found in 12% of the cases. In the right vagus nerve as a potential stimulation site to treat cardiac dysfunction [11,12], branching was observed in 22% of the donors. VNS on the right side alters atrial and ventricular function [11,12], but the mechanisms of action remain hypothetical. Recently, Seki and coworkers demonstrated that the human vagus nerve also contains sympathetic fibers with individual distribution patterns of catecholaminergic fibers [55]. The findings of Seki et al. [55] underline that the vagus nerve not only represents the parasympathetic part of the autonomic nervous system, but also includes sympathetic parts from a physiological point of view [56]. Furthermore, the occurrence of sympathetic fibers in the vagus nerve [55], published case reports on a Horner syndrome following VNS implantation [57,58] and our findings on CVN branching support the hypothesis that “no normal vagus nerve morphology and topography exists” [17]. As a consequence, VNS might be less safe than previously reported, with CVN branching as one of the potential causes for the side effects or the missing therapeutic efficiency of VNS [59].

### Limitations

This study is based on the anatomical findings in a limited number of cases with the surgical exposure expanded to the carotid sheath only. Since the donors were used for the student dissection course after we finished our study, we were not able to follow the branches distally, which is a major shortcoming of the given study. However, this approach is quite similar to the surgical one where the exposure for VNS is limited to the least necessary extent [17]. Here, vagus nerve branches are not traced back in the sense of “no-touch structures” to minimize the risk of injury. Nevertheless, we could demonstrate CVN branches in the carotid sheath macroscopically and provide histological proof that these branches consisted of nerve fibers. Moreover, the quality of the histological sections was limited by the ethanol-glycerin fixation technique, resulting in shrinkage of the samples. Another question that arises from our preparation is the exact anatomical location of the vagus nerve in the carotid sheath and potential connections to the sympathetic trunk of the neck [60], which should be addressed in future studies on the CVN.

### Summary and Outlook

The surgeon’s view of the vagus nerve has changed from a structure potential at risk in head and neck surgery to a target of nerve simulation. Beyond the established therapy of epilepsy and chronic heart failure, VNS is now applied to treat psychiatric conditions [9,10], headache [61–63] and even bronchial asthma [64]. Further targets of VNS are also related to inflammatory disorders, based on the cholinergic anti-inflammatory pathway [65,66]. An important safety issue is branching of the CVN in the carotid sheath, which may be related to the side effects or the missing therapeutic efficiency of VNS. To this end, preoperative ultrasonography may potentially help identify the vagus nerve position and branching [67]. Intra-operative neuromonitoring may further allow differentiating the main trunk of the CVN from potential branches, as frequently done in surgery of the thyroid gland [60].
